# Pathologic confirmation of valve thrombosis detected by four-dimensional computed tomography following valve-in-valve transcatheter aortic valve replacement

**DOI:** 10.21542/gcsp.2017.15

**Published:** 2017-06-30

**Authors:** Ambarish Gopal, Nathalia Ribeiro, John J. Squiers, Elizabeth M. Holper, Michael Black, Deepika Gopal, Molly Szerlip, Katherine B. Harrington, Srinivas Potluri, J. Michael DiMaio, David L. Brown, Paul A. Grayburn, Michael J. Mack, William T. Brinkman

**Affiliations:** The Heart Hospital Baylor Plano, 4716 Alliance Blvd, Suite 340, Plano, TX 75093, USA

## Abstract

A major concern regarding transcatheter aortic valve replacement (TAVR) is leaflet thrombosis. Four-dimensional computed tomography (4D-CT) is the preferred imaging modality to evaluate patients with suspected valve thrombosis. To date, the abnormal findings visualized by 4D-CT suggestive of leaflet thrombosis have lacked pathologic confirmation from a surgically explanted valve in a surviving patient. Herein, we provide pathologic confirmation of thrombus formation following surgical explantation of a thrombosed TAVR prosthesis that was initially identified by 4D-CT.

## Introduction

Valvular dysfunction and hemodynamic deterioration following transcatheter aortic valve replacement (TAVR) due to leaflet thrombosis is being reported with increasing frequency.^1–3^ This trend is the result of newfound scrutiny motivated by recent reports regarding restricted aortic valve prosthesis leaflet motion due to presumptive bioprosthetic valve leaflet thrombosis detected by four-dimensional, volume-rendered computed tomography (4D-CT).^4,5^ Importantly, however, 4D-CT imaging of suspected leaflet thrombosis has lacked pathologic confirmation from a surgically explanted TAVR valve to date.^1,3,6^ Herein, we provide pathologic confirmation of thrombus formation following surgical explantation of a thrombosed TAVR prosthesis that was initially visualized by 4D-CT.

## Case report

An 81-year-old male underwent surgical aortic valve replacement (SAVR) with 23 mm Magna valve (Edwards Lifesciences, Irvine, CA) and coronary artery bypass grafting in 2006 due to symptomatic severe aortic stenosis (AS) and coronary artery disease. In 2016, the patient was admitted to our hospital complaining of shortness of breath, orthopnea, and paroxysmal nocturnal dyspnea. He was diagnosed with acute on chronic combined systolic and diastolic heart failure. Transthoracic echocardiography (TTE) revealed low-flow/low-gradient severe AS (mean gradient [MG] 21mmHg; aortic valve area [AVA] 0.5 cm^2^). Given the patient’s high risk for redo-SAVR, valve-in-valve TAVR with a 23 mm Sapien 3 valve (Edwards Lifesciences) was performed via transfemoral access.

**Figure 1.**
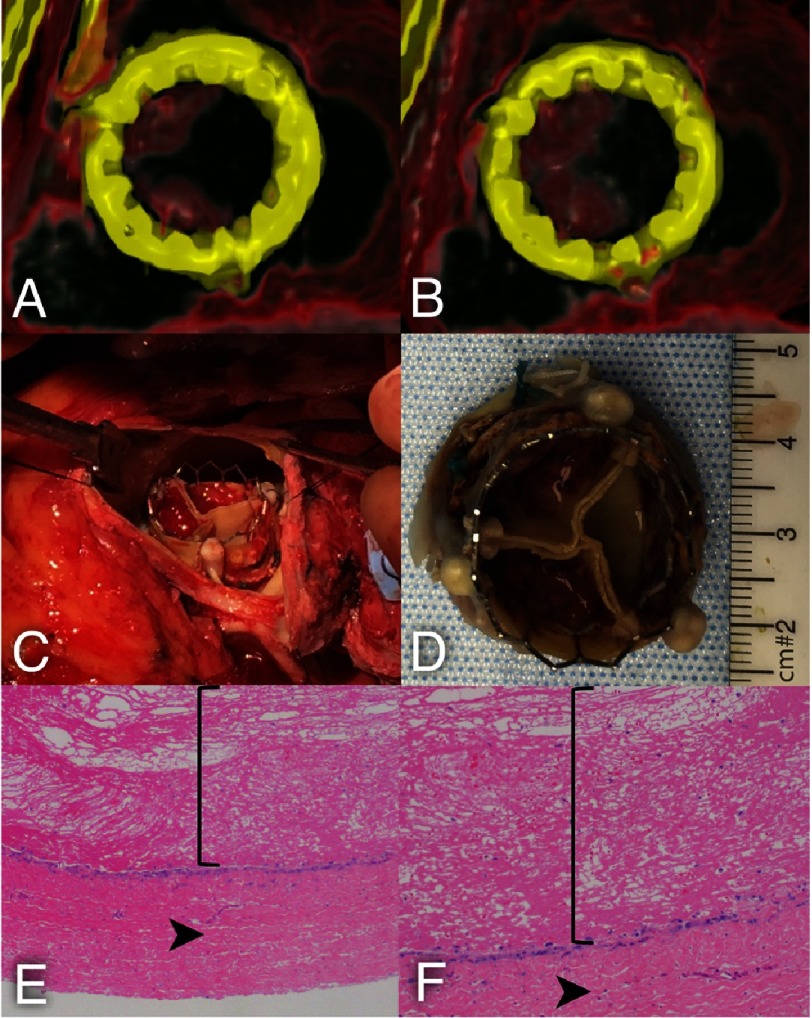
(A) Systolic short axis view of the TAVR prosthesis from the aortic side with thrombosis of two leaflets (white arrows). (B) Diastolic short axis view of the TAVR prosthesis from the aortic side with thrombosis of two leaflets (white arrows) with restricted leaflet mobility. (C) Thrombus was visualized *in situ*. (D) Explanted TAVR prosthesis with thrombosis of the two leaflets. (E, F) Histological analysis identified clot with organization at the periphery and endothelialized cells at the surface of the bioprosthetic valve material at 40× (E) and 100× (F) magnification. Straight bracket encompasses clot. Arrowhead identifies bioprosthetic valve material.

Post-TAVR, TTE demonstrated a successful implant profile with a mean gradient (MG) of 7 mmHg. The patient’s clinical condition worsened postoperatively, and he could not be weaned from inotropic support. TTE performed three days postoperatively demonstrated a MG of 29 mmHg. A 4D-CT was performed on a GE Revolution CT scanner (GE Healthcare, Waukesha, WI, USA) for suspected leaflet thrombosis. Data was analyzed and interpreted using Aquarius iNtuition (TeraRecon, Foster City, CA). The 4D-CT analysis revealed thrombus formation on two out of three neoleaflets of the TAVR prosthesis, which were both severely restricted in motion (Figure 1A–B).

Intravenous heparin therapy was initiated, but the patient continued to deteriorate clinically with increasing requirements for inotropic support. Two weeks after TAVR, a redo-SAVR with explantation of the TAVR prosthesis and replacement with a 25 mm Magna valve was successfully performed. In the operating room, thrombus was visualized on the same cusps identified by 4D-CT (Figure 1C–D). Histopathologic analysis identified clot formation and endothelialized cells at the surface of the bioprosthetic valve material (Figure 1E–F). The patient had a complicated postoperative course requiring tracheostomy and percutaneous endoscopic gastrostomy tube placement but was alive and recovering in the hospital on postoperative day 30.

## Comment

Predictors of valve thrombosis may be the absence of anticoagulation, valve-in-valve TAVR procedure, use of small (23-mm) valves, and increased body mass index.^2,6^ Our patient was not treated with anticoagulation after undergoing valve-in-valve TAVR with a 23-mm valve, and thus had several risk factors for thrombosis.

4D-CT imaging of TAVR (or SAVR) prostheses is indicated when increasing peak velocity and/or mean gradient by echocardiography suggests valvular dysfunction. Although restricted leaflet motion can be observed on transthoracic or transesophageal echocardiography, suspected leaflet thrombosis is best visualized by 4D-CT due to its superior spatial resolution.^1–6^ Although post-mortem autopsies have previously identified thrombus formation on TAVR prostheses, 4D-CT imaging findings suggestive of leaflet thrombosis have not been previously confirmed by pathologic analysis of a surgically explanted valve from a surviving patient.^1,3,6^ Importantly, this case provides pathologic confirmation that the abnormal findings identified by 4D-CT were in fact due to bioprosthetic valve leaflet thrombosis.

**Disclosure:** No conflicts of interest to disclose.
